# Passive and Active Triaxial Wall Mechanics in a Two-Layer Model of Porcine Coronary Artery

**DOI:** 10.1038/s41598-017-14276-1

**Published:** 2017-10-24

**Authors:** Yuan Lu, Hao Wu, Jiahang Li, Yanjun Gong, Jiahui Ma, Ghassan S. Kassab, Yong Huo, Wenchang Tan, Yunlong Huo

**Affiliations:** 1 Department of Cardiology, Peking University First Hospital, Beijing, China; 2 Department of Mechanics and Engineering Science, College of Engineering, Peking University, Beijing, China; 3 California Medical Innovations Institute, San Diego, USA; 4 School of Public Health, Peking University, Beijing, China; 5 Shenzhen Graduate School, Peking University, Shenzhen, China; 6 PKU-HKUST Shenzhen-Hongkong Institution, Shenzhen, China

## Abstract

Triaxial active and passive mechanical properties of coronary arteries are needed for understanding arterial mechanics in health and disease. The aim of the study was to quantify both active and passive strain energy functions in circumferential, axial and radial directions based on the experimental measurement. Moreover, a two-layer computational model was used to determine the transmural distribution of stresses and strains across the vessel wall. The first Piola-Kirchhoff stresses in the three normal directions had the approximate relationship as:$\,{T}_{\theta \theta }\cong 2{T}_{zz}\cong 5|{T}_{rr}|$. The two-layer model showed that circumferential Cauchy stresses increased significantly from the intima layer to the interface between media and adventitia layers (from ~80 to 160 kPa), dropped abruptly at the interface (from ~160 to <5 kPa), and increased slightly towards the outer boundary of the adventitia layer. In contrast, absolute values of radial Cauchy stress decreased continuously from the inner to outer boundaries of the vessel wall (from ~11 kPa to zero). Smooth muscle cell contraction significantly increased the ratio of radial to circumferential Cauchy stresses at the intima-media layer, which had the highest values at the intima layer.

## Introduction

Coronary artery disease (CAD) is the major cause of mortality and morbidity in the World^1,2^. Although substantial studies have emphasized the role of hemodynamic stimuli on CAD^3,4^, the mechanical properties of coronary artery wall are fundamental for understanding the basic mechanisms of these diseases^5–7^. Since the intima layer does not contribute to mechanical properties of the vessel wall^8^, the arterial mechanics is generally performed in a two-layer model; i.e., intima-media (IM) and adventitia layers. Collagen fibers and elastin networks in the entire vessel wall determine passive mechanical properties and smooth muscle cells (SMCs) in the IM layer dictate active properties.

Uniaxial studies of circumferential passive and active mechanical properties of the vessel wall are very prevalent^9–16^ while a few biaxial models have been proposed in the circumferential and axial directions^17–19^. The transmural changes of radial stresses and strains can also contribute to vascular function (e.g., SMC migration) in response to abnormal hemodynamic stimuli (e.g., high blood pressure). To our knowledge, there is still no three-dimensional (3D) mechanical analysis of both active and passive stress-strain relations in a two-layer model of coronary arteries.

The objective of this study was to determine triaxial (circumferential, axial and radial) active and passive mechanical properties in the entire vessel wall and IM layer of porcine coronary artery. Based on the constitutive relation, a two-layer model was developed to compute the transmural distribution of circumferential and radial stresses and strains across the vessel wall. A large-scale global search algorithm (i.e., genetic algorithm) was developed to quantify material constants of 3D active and passive strain energy functions based on the experimental measurements^17,18^. The significance, implications and limitations of the study are discussed to improve the understanding of coronary arterial mechanics.

## Methods

### Existing Data

Huo *et al*. have previously reported passive and active mechanical tests in the intact vessel wall and IM layer of porcine right coronary arteries (RCA)^17,18^. Briefly, 5 farm pigs weighing 28 ± 5 Kg were used for mechanical test in the intact vessel wall^18^. RCA was isolated from the heart and cannulated on both ends with the connector in an organ bath containing HEPES PSS at 37 °C and aerated with 95% O_2_-5% CO_2_. The 37 °C PSS in bath was replaced by 60 mM K^+^ PSS at the same temperature. After the vessel was preconditioned several times to obtain reproducible mechanical data at axial stretch ratio (*λ*_*z*_) of 1.3 and 1.5, transmural pressures were varied from 20 to 200 mmHg by an increment of 20 mmHg. The changes of diameter and axial force were recorded. After the active test, the organ bath was filled with Ca^2+^-free Krebs solution to relax the RCA. After removal of vasoactivity, the passive pressure-diameter-axial force relation of the vessel was determined for ${\lambda }_{z}$ = 1.3 and 1.5 similar to the active protocol. Moreover, 6 farm pigs weighing 30 ± 5 Kg were used for the study of RCA IM layer^17^. After the adventitia of RCA was carefully dissected with the aid of a stereomicroscope to ensure that the IM layer remained entire, we repeated the same procedure as that in the entire vessel wall. In the study, superscripts “IM” and “A” refer to the IM and adventitia layers, respectively.

### Mathematical models

Fung’s 3D model without shear deformation^20–22^ was selected to characterize passive mechanical properties and a previous 2D active model^17,18^ is extended to describe active mechanical properties. Hence, 3D passive and active strain energy functions (${W}_{passive}$ and $\,{W}_{active}$, respectively) are written as:

1 $$\begin{array}{rcl}{W}_{passive} & = & \frac{1}{2}{C}_{1}[{e}^{Q}-1]\\ {W}_{active} & = & {C}_{2}[Erf(Q^{\prime} )-1]\\ {W}_{total} & = & {W}_{passive}+{W}_{active}\end{array}$$

where $Q={a}_{1}{E}_{\theta \theta }^{2}+{a}_{2}{E}_{zz}^{2}+{a}_{3}{E}_{rr}^{2}+2{a}_{4}{E}_{\theta \theta }{E}_{zz}+2{a}_{5}{E}_{zz}{E}_{rr}+2{a}_{6}{E}_{rr}{E}_{\theta \theta }$, $\,Q^{\prime} =\frac{{\lambda }_{\theta }}{{b}_{1}}+\frac{{\lambda }_{z}}{{b}_{2}}+\frac{{\lambda }_{r}}{{b}_{3}}-b^{\prime}$, and $b^{\prime} =\frac{{b}_{4}}{{b}_{1}}+\frac{{b}_{5}}{{b}_{2}}+$ $\frac{{b}_{6}}{{b}_{3}}$; ${C}_{1},\,{C}_{2},\,{a}_{1}-{a}_{6}$ and ${b}_{1}-{b}_{6}$ are material constants; $Erf(X)$ is the Gauss error function; ${E}_{\theta \theta },{E}_{zz}$, and *E*_*rr*_ are circumferential, axial and radial Green strains, respectively; and ${\lambda }_{\theta }$, ${\lambda }_{z}$ and ${\lambda }_{r}$ represent the corresponding stretch ratios. Here, ${\lambda }_{\theta }=\frac{l}{{l}_{0}}=\sqrt{2{E}_{\theta \theta }+1},\,{\lambda }_{z}=\frac{L}{{L}_{0}}=\sqrt{2{E}_{zz}+1}$ and ${\lambda }_{r}=\frac{1}{{\lambda }_{\theta }{\lambda }_{z}}$, where *l* and *l*_0_ are circumferential lengths in loaded and zero-stress states; and *L* and *L*_0_ are axial lengths in loaded and no-load states (the axial length at the no-load state is assumed equal to that at the zero-stress state)^9^. Although an actively contracting system is not necessarily described by a strain-energy function, this approach is used here to obtain an empirical fit to the observed active mechanical properties. Moreover, the first Piola-Kirchhoff (1^st^ PK) stresses (${T}_{\theta \theta }$, ${T}_{zz}$ and ${T}_{rr}$) are computed from the 3D strain energy functions theoretically (see Appendix A).

### Determination of material constants

Passive material constants are determined by minimizing the square of difference between theoretical and experimental values of passive 1^st^ PK stresses as:

2 $$Erro{r}_{passive}={\sum }_{n=1}^{N}[\begin{array}{c}{({T}_{\theta \theta passive}^{theory}-{T}_{\theta \theta passive}^{experiment})}^{2}\\ +{({T}_{zzpassive}^{theory}-{T}_{zzpassive}^{experiment})}^{2}\\ +{({T}_{rrpassive}^{theory}-{T}_{rrpassive}^{experiment})}^{2}\end{array}]$$

where *N* is the total number of experimental points when a vessel is at the maximal vasodilation; and ${T}_{\theta \theta \,passive\,}^{experiment}=\frac{P{r}_{i}}{{\lambda }_{\theta }h}$, where *P*, ${r}_{i}=\sqrt{{r}_{o}^{2}-\frac{{A}_{0}}{\pi {\lambda }_{z}}}$, and $h={r}_{o}-{r}_{i}$ refer to transmural pressure, inner radius, and wall thickness in loaded state, respectively (*r*_*o*_ is the outer radius and *A*_0_ the wall area in no-load state), ${T}_{zz\,passive}^{experiment}=\frac{1}{{\lambda }_{z}}[\frac{F}{\pi ({r}_{o}^{2}-{r}_{i}^{2})}+\frac{P{r}_{i}^{2}}{h({r}_{o}+{r}_{i})}]$ (*F*: the axial force), and ${T}_{rr\,passive}^{experiment}=\frac{-{r}_{i}P}{({r}_{o}+{r}_{i}){\lambda }_{r}}$. Similar to Eq. [2], the square of difference between theoretical and experimental values of total 1^st^ PK stresses is given as:

3 $$Erro{r}_{total}={\sum }_{n=1}^{N}[\begin{array}{c}{({T}_{\theta \theta active}^{theory}+{T}_{\theta \theta passive}^{theory}-{T}_{\theta \theta total}^{experiment})}^{2}\\ +{({T}_{zzactive}^{theory}+{T}_{zzpassive}^{theory}-{T}_{zztotal}^{experiment})}^{2}\\ +{({T}_{rractive}^{theory}+{T}_{rrpassive}^{theory}-{T}_{rrtotal}^{experiment})}^{2}\end{array}]$$

where *N* is the total number of experimental points when a vessel is at the maximal vasoconstriction, based on which ${T}_{\theta \theta \,total\,}^{experiment}$, ${T}_{rr\,total}^{experiment}$, and ${T}_{zz\,total}^{experiment}$ are determined.

A large-scale global search algorithm, genetic algorithm (GA)^23^, was used to minimize the error function as expressed by Eqs [2] and [3] given that ${C}_{1},{C}_{2},{a}_{1} \sim {a}_{6},{b}_{1} \sim {b}_{3} > 0,b^{\prime}  > 0$ and ${a}_{4}^{2}+{a}_{5}^{2}+{a}_{6}^{2}-{a}_{1}{a}_{2}-{a}_{2}{a}_{3}$ $-{a}_{1}{a}_{3} > 0$ to satisfy the hyperelasticity condition^24,25^. Briefly, a MATLAB code was developed to determine material constants based on the MATLAB Genetic Algorithm Toolbox, similar to previous studies^26,27^. A number of parameters were selected, including the size of the population, probability of crossover, and mutation; scale for mutation and Tournament probability; and number of generations. The search was initiated for the best material constants obtained from the Marquardt-Levenberg (M-L) method. The following search steps were like a previous study^27^.

### A two-layer computational model

Similar to a previous study^17^, a two-layer model (see Appendix B) was demonstrated to compute the transmural distribution of stresses and stretches across the vessel wall at passive and active states. Material constants of the 3D passive strain energy function for IM and adventitia layers as well as stress-free geometry were consistent with those in Tables A1 and A2 of ref.^17^. Material constants (*C*_2_ = 28.92, *b*_1_ = 0.43, *b*_2_ = 1.62, *b*_3_ = 4.38, and *b*′ = 4.87) of the 3D active strain energy function in the IM layer were determined by the optimal fit of all six RCA IM layers. The transmural distribution of stresses was computed at healthy and pressure-overload states.

### Data Analysis

ANOVA (SigmaStat 3.5) was used to compare 3D experimental and theoretical results of passive and active mechanical properties in circumferential, axial and radial directions, where p value <0.05 represented the statistically significant difference. Moreover, we carried out the sensitivity analysis of material constants ${b}_{1} \sim {b}_{3}$ in the 3D active strain energy function to determine the effects on the active 1^st^ PK stresses, which was compared with the 2D active model^17,18^.

## Results

**Figure 1:**
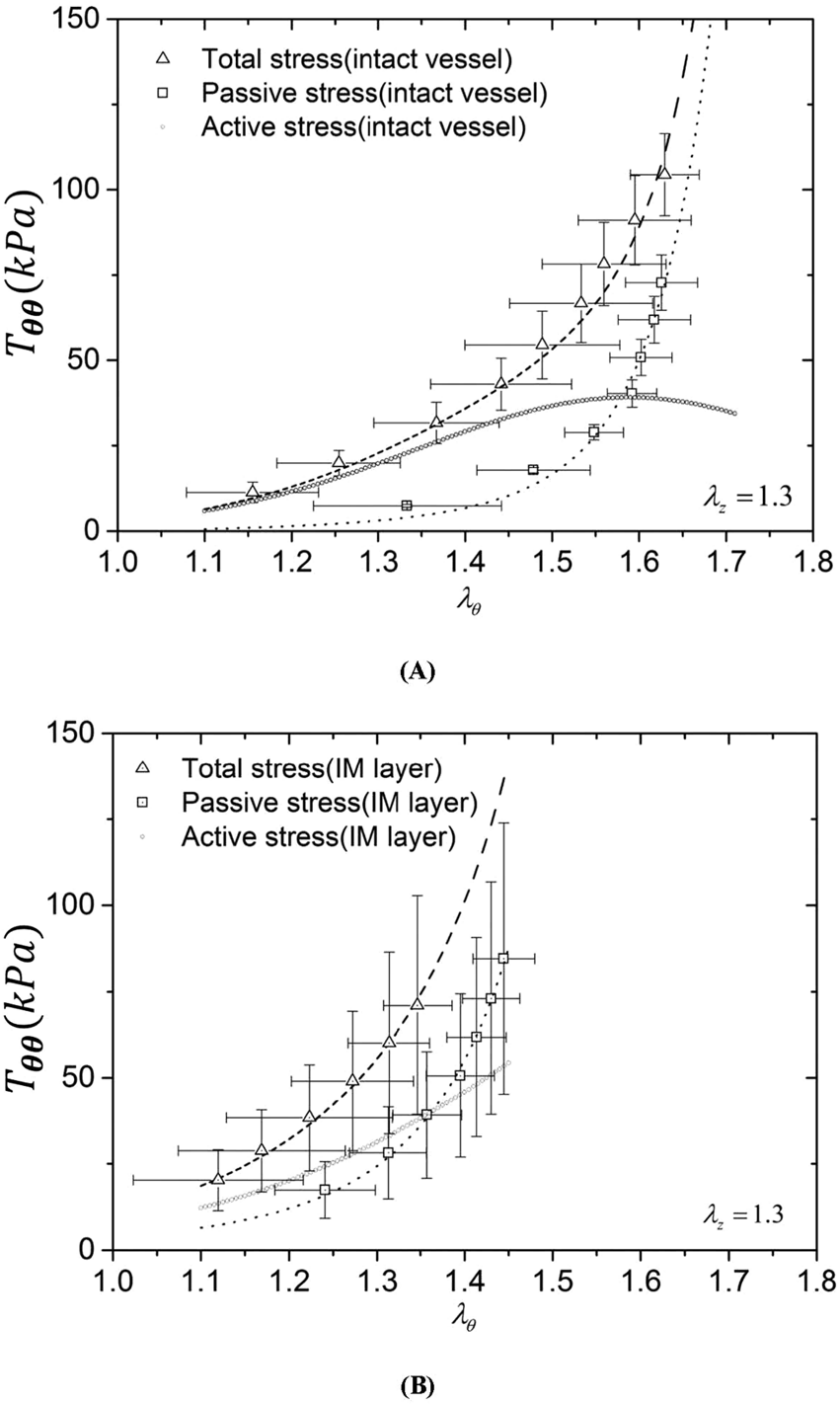
Circumferential first Piola-Kirchhoff stress (${T}_{\theta \theta }$) as a function of circumferential stretch ratio (${\lambda }_{\theta }$) with an interval of 0.05 from 1.1 to 1.7 for the entire vessel wall (**A**) and from 1.1 to 1.45 for the IM layer (**B**). Total stress (□ marks with error bars of SD): ${(\frac{P{r}_{i}}{{\lambda }_{\theta }h})}_{total}$ at vasoconstriction; passive stress (△ marks with error bars of SD): ${(\frac{P{r}_{i}}{{\lambda }_{\theta }h})}_{passive}$ at vasodilation. The dash line, dash dot line and dash line in round point refer to theoretical passive, total and active stresses, respectively. The plots show the averaged values over the entire wall or IM layer thickness.

**Figure 2:**
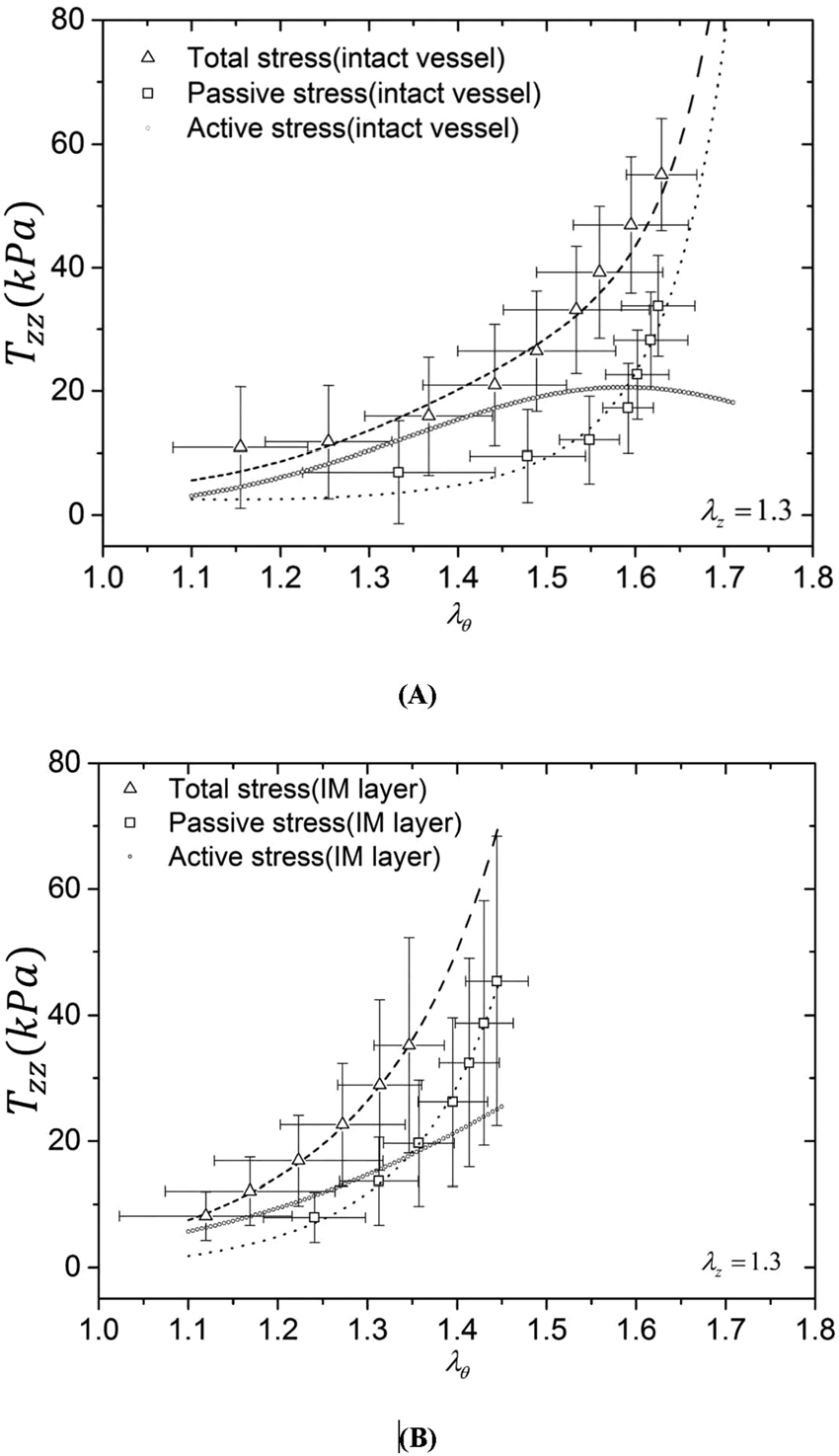
Axial first Piola-Kirchhoff stress (${T}_{zz}$) as a function of circumferential stretch ratio (${\lambda }_{\theta }$) with an interval of 0.05 from 1.1 to 1.7 for the entire vessel wall (**A**) and from 1.1 to 1.45 for the IM layer (**B**). Total stress (□ marks with error bars of SD): $\frac{1}{{\lambda }_{z}}{[\frac{F}{\pi ({r}_{o}^{2}-{r}_{i}^{2})}+\frac{P{r}_{i}^{2}}{h({r}_{o}+{r}_{i})}]}_{total}$ at vasoconstriction; passive stress (△ marks with error bars of SD): $\frac{1}{{\lambda }_{z}}{[\frac{F}{\pi ({r}_{o}^{2}-{r}_{i}^{2})}+\frac{P{r}_{i}^{2}}{h({r}_{o}+{r}_{i})}]}_{passive}$ at vasodilation. The dash line, dash dot line and dash line in round point refer to theoretical passive, total and active stresses, respectively. The plots show the averaged values over the entire wall or IM layer thickness.

**Figure 3:**
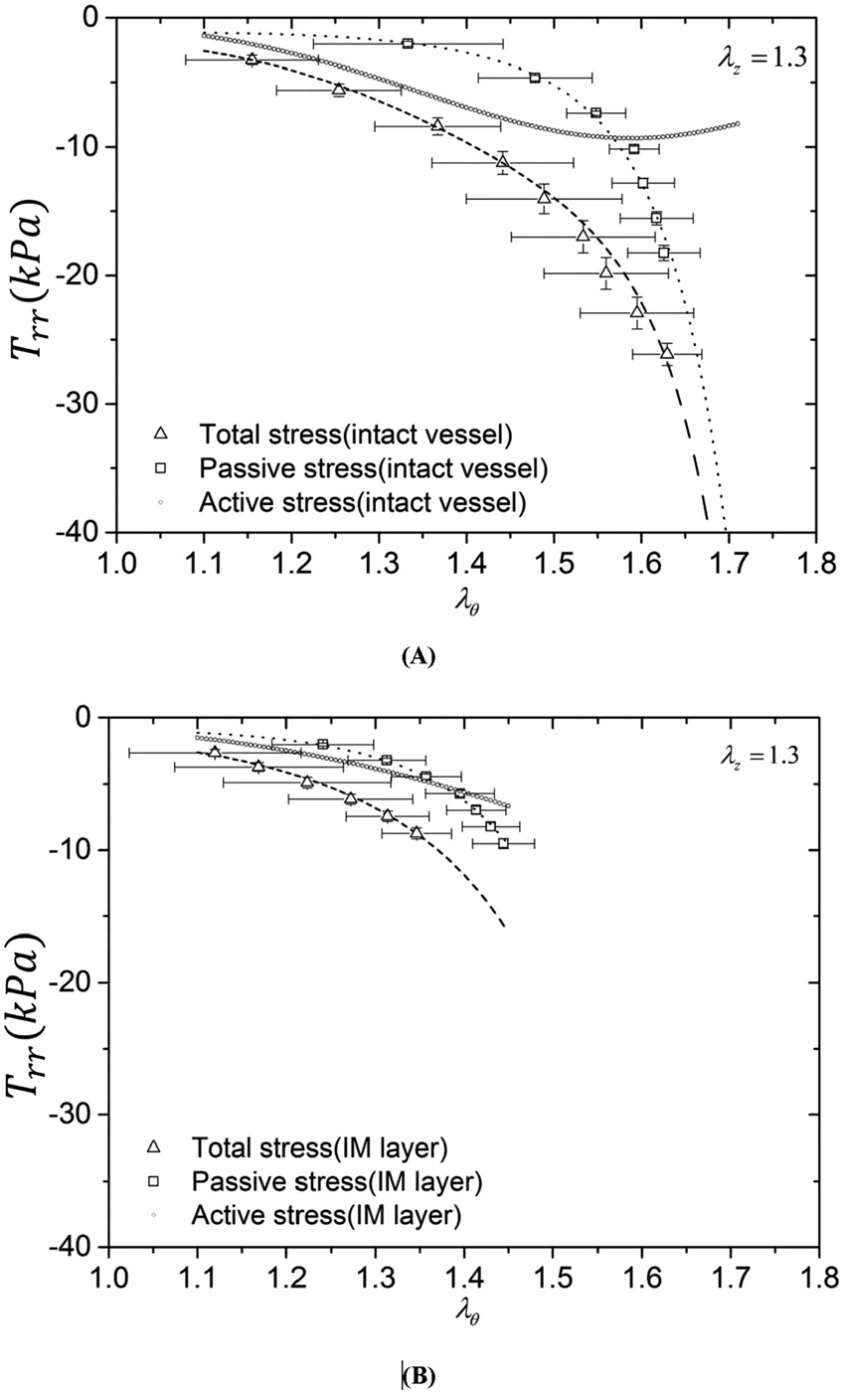
Radial first Piola-Kirchhoff stress (${T}_{rr}$) as a function of circumferential stretch ratio (${\lambda }_{\theta }$) with an interval of 0.05 from 1.1 to 1.7 for the entire vessel wall (**A**) and from 1.1 to 1.45 for the IM layer (**B**). Total stress (□ marks with error bars of SD): ${[\frac{-{r}_{i}P}{({r}_{o}+{r}_{i}){\lambda }_{r}}]}_{total}$ at vasoconstriction; passive stress (Δ marks with error bars of SD): ${[\frac{-{r}_{i}P}{({r}_{o}+{r}_{i}){\lambda }_{r}}]}_{passive}$ at vasodilation. The dash line, dash dot line and dash line in round point refer to theoretical passive, total and active stresses, respectively. The plots show the averaged values over the entire wall or IM layer thickness.

**Figure 4:**
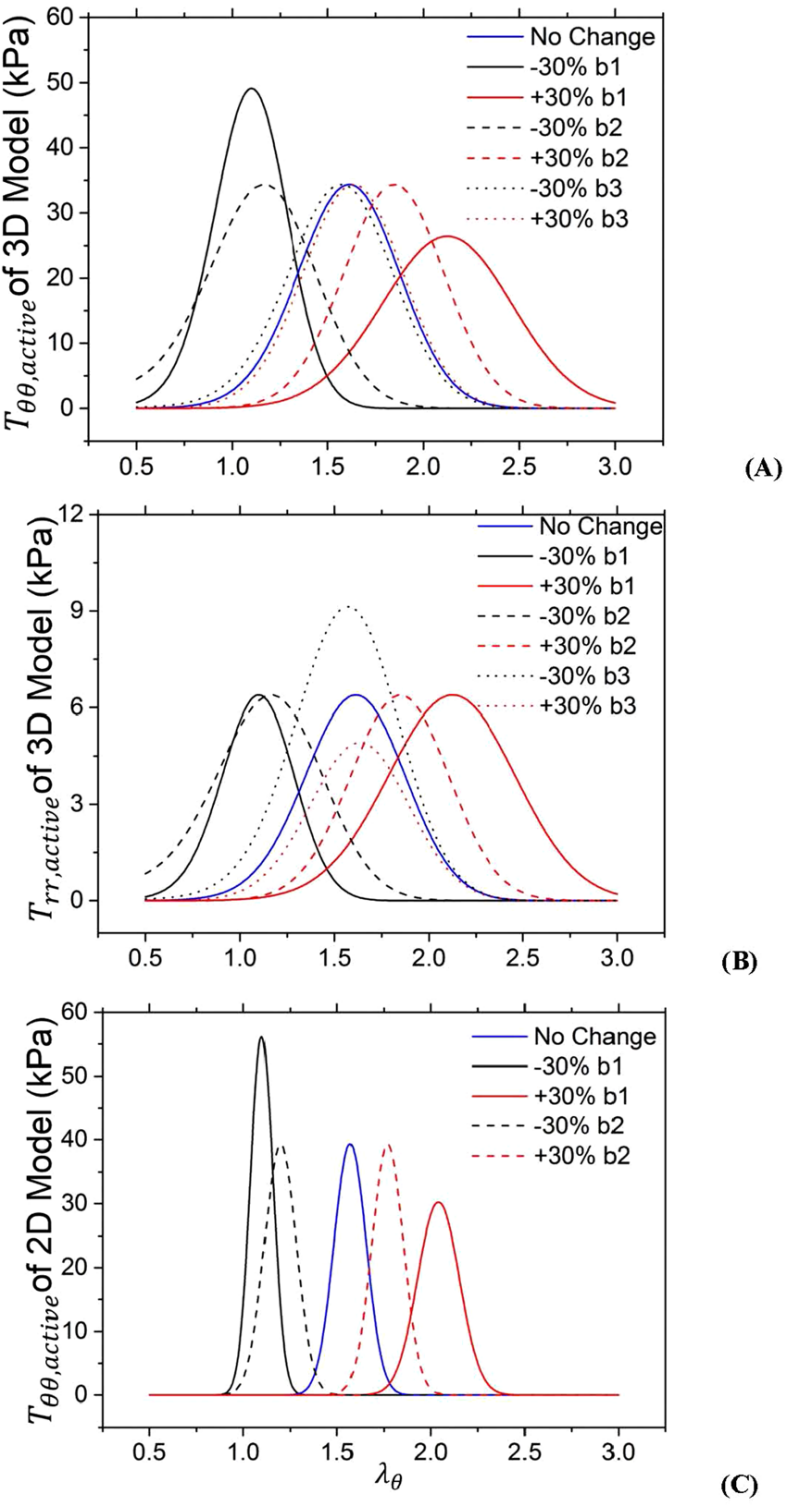
(**A**,**B**) Sensitivity analysis of material constants ${b}_{1} \sim {b}_{3}$ in the 3D active strain energy function for active 1^st^ PK stresses ${T}_{\theta \theta ,active}$ (**A**) and ${T}_{rr,active}$ (**B**), where *b*_1_ = 0.35, *b*_2_ = 0.47, and *b*_3_ = 1.89 (i.e., material constants determined by the optimal fit of 5 entire RCA walls); (**C**) Sensitivity analysis of material constants *b*_1_ and *B*_2_ in the 2D active strain energy function (ref.^17^) for active 1^st^ PK stress ${T}_{\theta \theta ,active}$, where *b*_1_ = 0.12 and *b*_2_ = 0.18. The plots show the averaged values over the entire wall thickness.

Tables 1 and 2 list material constants of Fung’s passive and K^+^-induced active 3D strain energy functions in the entire vessel wall and IM layer of each RCA, respectively. Good agreement is demonstrated between experimental measurements and theoretical predictions with parameters fitted by the genetic algorithm. Figures 1A, 2A and 3A show circumferential, axial and radial 1^st^ PK stresses, respectively, as a function of ${\lambda }_{\theta }$ with an interval of 0.05 from 1.1 to 1.7 for the entire vessel wall. The square and triangle marks with error bars of SD (averaged with all vessels) represent experimental passive and total stresses, respectively. The dash line, dash dot line and dash line in round point refer to theoretical passive, total and active stresses. The 1^st^ PK stress decreases in a sequence of circumferential, axial and radial directions, ${T}_{\theta \theta passive} > {T}_{zzpassive} > |{T}_{rrpassive}|$, ${T}_{\theta \theta active} > {T}_{zzactive} > |{T}_{rractive}|$, and ${T}_{\theta \theta total} > {T}_{zztotal} > |{T}_{rrtotal}|$ (${T}_{\theta \theta }\cong 2{T}_{zz}\cong 5|{T}_{rr}|)$. Figures 1B, 2B and 3B show the three 1^st^ PK stresses as a function of ${\lambda }_{\theta }$ with an interval of 0.05 from 1.1 to 1.45 for the IM layer, which presents that ${T}_{\theta \theta }^{IM} > {T}_{zz}^{IM} > |{T}_{rr}^{IM}|$ in active and passive states similar to the entire vessel wall. At a circumferential stretch ratio (e.g., at ${\lambda }_{\theta }$ of 1.4), the passive 1^st^ PK stresses have higher values in the IM layer than the entire vessel wall despite similar active values. Furthermore, Fig. 4A and B show the sensitivity analysis of material constants ${b}_{1} \sim {b}_{3}$ in the 3D active strain energy function for active 1^st^ PK stresses ${T}_{\theta \theta ,active}$ and ${T}_{rr,active}$, respectively, in the vessel wall. Figure 4C presents the corresponding sensitivity analysis in the 2D active model. The plots in Figs 1–4 show the averaged values over the entire wall or IM layer thickness.

**Table 1:** Material constants of Fung’s passive and K+-induced active 3D strain energy functions in the entire vessel wall.

Animal No.	*C*_1_	*a*_1_	*a*_2_	*a*_3_	*a*_4_	*a*_5_	*a*_6_	${{\boldsymbol{R}}}_{{{\boldsymbol{T}}}_{{\boldsymbol{\theta }}{\boldsymbol{\theta }},{\boldsymbol{passive}}}}^{{\bf{2}}}$	${{\boldsymbol{R}}}_{{{\boldsymbol{T}}}_{{\boldsymbol{zz}},{\boldsymbol{passive}}}}^{{\bf{2}}}$	${{\boldsymbol{R}}}_{{{\boldsymbol{T}}}_{{\boldsymbol{rr}},{\boldsymbol{passive}}}}^{{\bf{2}}}$
Heart 1	5.70	1.96	6.60	6.11	1.72	6.70	1.64	0.964	0.953	0.957
Heart 2	3.64	3.02	9.60	5.70	1.19	8.20	1.61	0.974	0.968	0.968
Heart 3	9.30	2.44	2.13	1.71	3.51	3.99	4.16	0.938	0.902	0.935
Heart 4	4.72	4.81	3.30	8.63	10.30	5.02	2.81	0.921	0.932	0.937
Heart 5	8.10	3.02	1.19	1.61	11.22	2.36	0.50	0.902	0.878	0.941
Mean	6.29	3.05	4.56	4.75	5.59	5.25	2.15	0.940	0.927	0.948
SD	2.35	1.08	3.48	3.04	4.81	2.28	1.39	0.03	0.03	0.01
**Animal No**.	***C***_**2**_	***b***_**1**_	***b***_**2**_	***b***_**3**_	***b*** **′**	${{\boldsymbol{R}}}_{{{\boldsymbol{T}}}_{{\boldsymbol{\theta }}{\boldsymbol{\theta }},{\boldsymbol{active}}}}^{{\bf{2}}}$	${{\boldsymbol{R}}}_{{{\boldsymbol{T}}}_{{\boldsymbol{zz}},{\boldsymbol{active}}}}^{{\bf{2}}}$	${{\boldsymbol{R}}}_{{{\boldsymbol{T}}}_{{\boldsymbol{rr}},{\boldsymbol{active}}}}^{{\bf{2}}}$		
Heart 1	17.6	0.54	1.08	2.40	5.45	0.908	0.938	0.914		
Heart 2	12.3	0.25	0.41	1.02	9.43	0.912	0.936	0.951		
Heart 3	7.78	0.18	0.44	0.68	11.8	0.981	0.939	0.941		
Heart 4	11.1	0.79	1.21	3.72	4.06	0.920	0.899	0.902		
Heart 5	15.1	0.48	0.99	1.62	5.63	0.918	0.975	0.927		
Mean	12.8	0.45	0.83	1.89	7.28	0.928	0.937	0.927		
SD	3.76	0.24	0.37	1.21	3.22	0.03	0.03	0.02

**Table 2:** Material constants of Fung’s passive and K^+^-induced active 3D strain energy functions in the IM layer.

Animal No.	*C*_1_	*a*_*1*_	*a*_2_	*a*_3_	*a*_4_	*a*_5_	*a*_6_	${{\boldsymbol{R}}}_{{{\boldsymbol{T}}}_{{\boldsymbol{\theta }}{\boldsymbol{\theta }},{\boldsymbol{passive}}}}^{{\bf{2}}}$	${{\boldsymbol{R}}}_{{{\boldsymbol{T}}}_{{\boldsymbol{zz}},{\boldsymbol{passive}}}}^{{\bf{2}}}$	${{\boldsymbol{R}}}_{{{\boldsymbol{T}}}_{{\boldsymbol{rr}},{\boldsymbol{passive}}}}^{{\bf{2}}}$
Heart 1	6.29	2.79	1.64	5.49	2.50	3.20	2.11	0.999	0.999	0.999
Heart 2	4.58	3.51	2.29	4.1	4.22	2.72	2.93	0.992	0.993	0.992
Heart 3	7.21	5.23	2.56	5.67	4.21	4.56	2.44	0.998	0.990	0.986
Heart 4	2.38	4.45	3.28	3.26	3.84	3.52	1.26	0.950	0.953	0.946
Heart 5	4.41	6.63	5.68	4.13	4.63	5.72	6.19	0.908	0.891	0.910
Heart 6	5.92	7.16	5.21	3.02	5.99	6.26	4.99	0.923	0.928	0.922
Mean	5.13	4.96	3.44	4.28	4.23	4.33	3.32	0.962	0.959	0.959
SD	1.71	1.51	1.55	1.04	0.84	1.48	1.99	0.04	0.04	0.04
**Animal No**.	***C***_**2**_	***b***_**1**_	***b***_**2**_	***b***_**3**_	***b*** **′**	${{\boldsymbol{R}}}_{{{\boldsymbol{T}}}_{{\boldsymbol{\theta }}{\boldsymbol{\theta }},{\boldsymbol{active}}}}^{{\bf{2}}}$	${{\boldsymbol{R}}}_{{{\boldsymbol{T}}}_{{\boldsymbol{zz}},{\boldsymbol{active}}}}^{{\bf{2}}}$	${{\boldsymbol{R}}}_{{{\boldsymbol{T}}}_{{\boldsymbol{rr}},{\boldsymbol{active}}}}^{{\bf{2}}}$		
Heart 1	49.8	0.55	1.13	4.66	4.79	0.997	0.991	0.996		
Heart 2	47.5	0.43	0.92	1.60	6.28	0.995	0.997	0.996		
Heart 3	42.1	0.49	1.05	3.14	5.23	0.997	0.996	0.998		
Heart 4	40.3	0.64	1.32	7.37	3.96	0.955	0.989	0.942		
Heart 5	40.2	0.34	0.72	5.77	5.94	0.964	0.979	0.959		
Heart 6	45.2	0.58	1.22	3.20	4.86	0.966	0.982	0.962		
Mean	44.2	0.51	1.06	4.29	5.18	0.979	0.989	0.976		
SD	3.63	0.10	0.20	1.90	0.77	0.02	0.01	0.02

**Figure 5:**
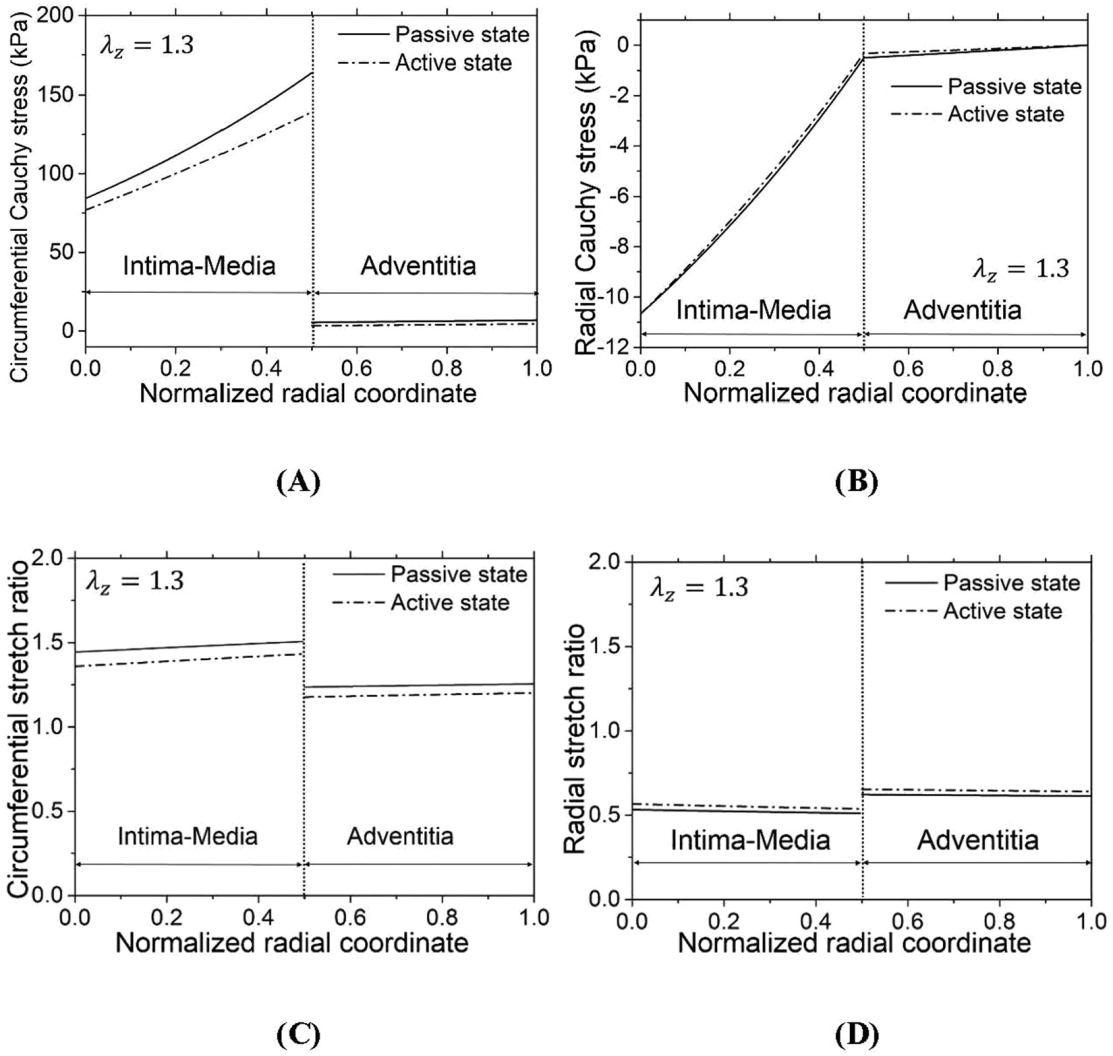
(**A**,**B**) Transmural distribution of circumferential Cauchy stress ${\sigma }_{\theta }$ (**A**) and radial Cauchy stress ${\sigma }_{r}$ (**B**) across the normalized vessel wall; (**C**,**D**) Transmural distribution of circumferential stretch ratio ${\lambda }_{\theta }$ (**C**) and radial stretch ratio ${\lambda }_{r}$ (**D**) across the normalized vessel wall at ${\lambda }_{z}$ of 1.3 and transmural pressure of 80 mmHg. The results were computed when the 3D active strain energy function was applied to the IM layer and the 3D passive strain energy function was applied to the entire vessel wall (*h* = 0.22 mm with the thickness of IM layer equal to $\frac{h}{2}$).

Figure 5A and B show the transmural distribution of circumferential and radial Cauchy stresses, respectively, across the normalized vessel wall at ${\lambda }_{z}$ of 1.3, transmural pressure of 80 mmHg, and wall thickness of 0.22 mm. Circumferential Cauchy stresses between IM and adventitia layers are discontinuous with significantly higher values in the IM layer. Figure 5C and D show the transmural distribution of circumferential and radial stretch ratios, both of which present discontinuity at the interface between IM and adventitia layers. In contrast, radial Cauchy stresses are continuous across the entire vessel wall. In particular, K^+^-induced vasoconstriction significantly decreases circumferential Cauchy stresses (~15%, p value < 0.05) in the IM layer, but has relatively negligible effects on radial Cauchy stresses, which results in an increased ratio of radial to circumferential Cauchy stresses in the IM layer.

**Figure 6:**
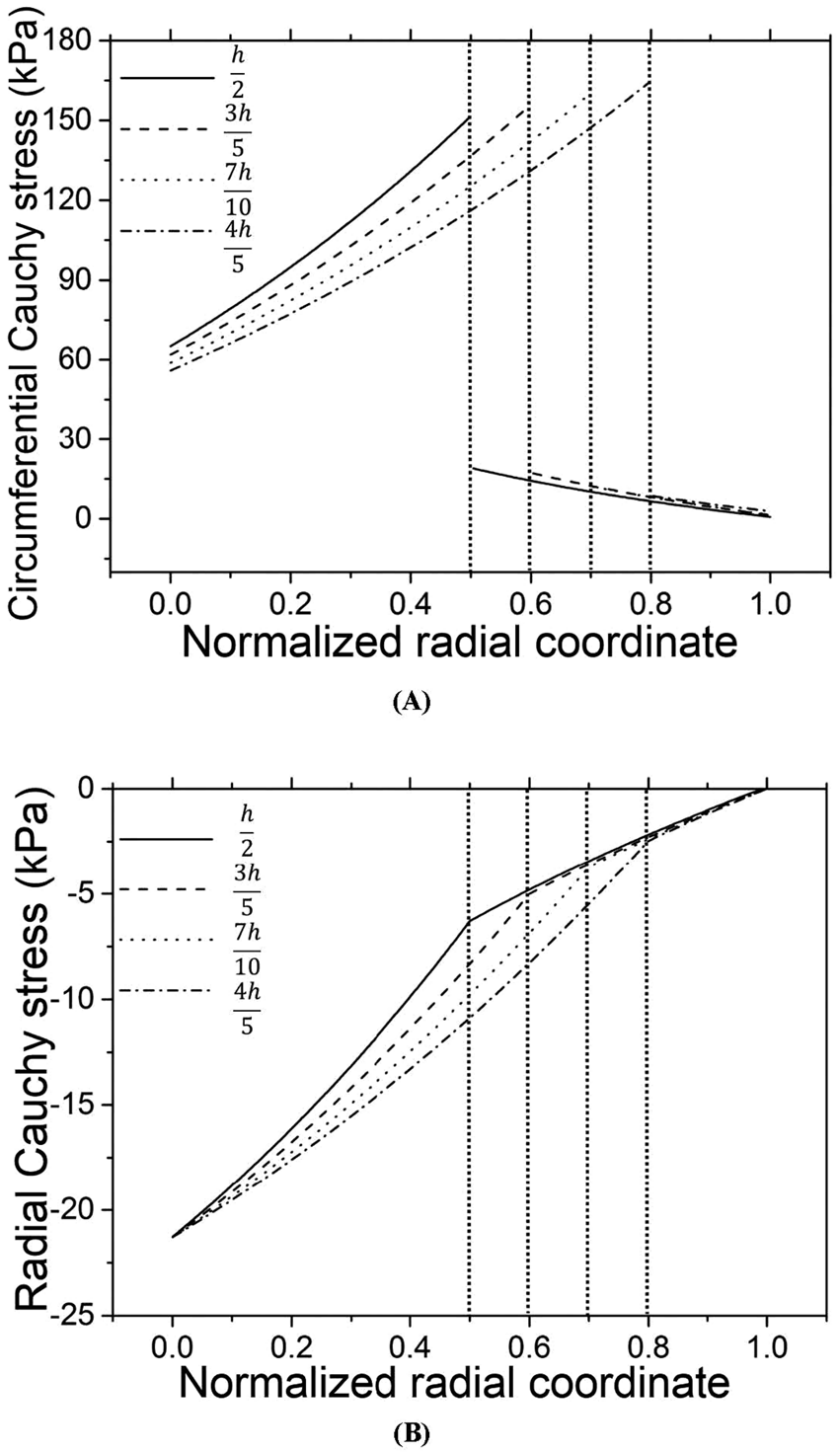
Transmural distribution of circumferential Cauchy stress ${\sigma }_{\theta }$ (**A**) and radial Cauchy stress ${\sigma }_{r}$ (**B**) across the normalized vessel wall under active state at ${\lambda }_{z}$ of 1.3 and transmural pressure of 160 mmHg. Corresponding to Fig. 5, the results were computed when the 3D active strain energy function was applied to the IM layer and the 3D passive strain energy function was applied to the entire vessel wall (*h* = 0.44 mm with the thickness of IM layer equal to $\frac{h}{2}$, $\frac{3h}{5}$, $\frac{7h}{10}$, and $\frac{4h}{5}$).

We investigated the effects of pressure overload on the transmural distribution of Cauchy stresses. Figure 6A and B show the transmural distribution of circumferential and radial Cauchy stresses, respectively, across the normalized vessel wall under the active state. The transmural pressure and wall thickness increase to 160 mmHg and 0.44 mm (twofold increase from those in Fig. 5), respectively, to mimic pressure overload. This significantly increases the radial Cauchy stresses despite the relatively unchanged circumferential values. Moreover, an increase in the thickness ratio of IM layer to the entire vessel wall leads to an increase and decrease of radial and circumferential Cauchy stresses, respectively.

**Figure 7:**
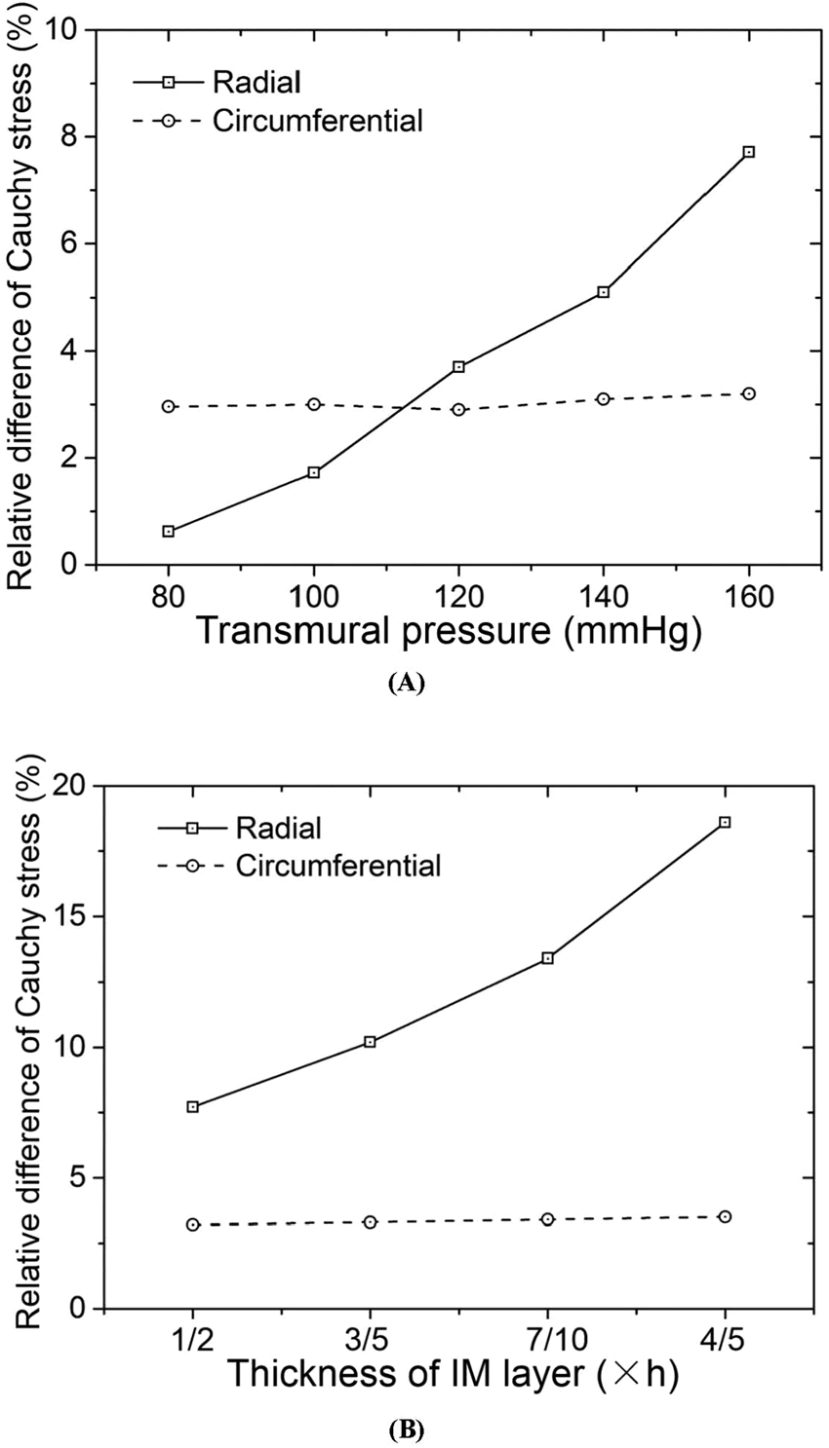
(**A**) The relative difference of computational Cauchy stresses from the two-layer model with the 2D (ref.^17^) or 3D (Eq. 1) active strain energy function in the IM layer as well as the same 3D passive strain energy function in the entire vessel wall (i.e., $|\frac{{\sigma }_{\theta }^{3D}-{\sigma }_{\theta }^{2D}}{{\sigma }_{\theta }^{3D}}|$ and $|\frac{{\sigma }_{r}^{3D}-{\sigma }_{r}^{2D}}{{\sigma }_{r}^{3D}}|$ at the interface between media and adventitia layers) as a function of transmural pressures at ${\lambda }_{z}$ of 1.3. The entire vessel wall thickness (the thickness of IM layer equal to *h*/2 in correspondence with Fig. 5) changes with the transmural pressure to maintain the averaged circumferential Cauchy stress over the entire wall thickness given the uniform circumferential stress hypothesis; (**B**) The relative difference of computational Cauchy stresses from the two-layer model with the 2D (ref.^17^) or 3D (Eq. 1) active strain energy function in the IM layer as well as the same 3D passive strain energy function in the entire vessel wall (i.e., $|\frac{{\sigma }_{\theta }^{3D}-{\sigma }_{\theta }^{2D}}{{\sigma }_{\theta }^{3D}}|$ and $|\frac{{\sigma }_{r}^{3D}-{\sigma }_{r}^{2D}}{{\sigma }_{r}^{3D}}|$ at the interface between media and adventitia layers) as the thickness of IM layer varies from *h*/2 to 4*h*/5 corresponding to Fig. 6.

We demonstrated a comparison of computational Cauchy stresses from the two-layer model with the 2D or 3D active strain energy function in the IM layer as well as the same 3D passive strain energy function in the vessel wall. Figure 7A shows the relative difference of circumferential and radial Cauchy stresses (i.e., $|\frac{{\sigma }_{\theta }^{3D}-{\sigma }_{\theta }^{2D}}{{\sigma }_{\theta }^{3D}}|$ and $|\frac{{\sigma }_{r}^{3D}-{\sigma }_{r}^{2D}}{{\sigma }_{r}^{3D}}|$ at the interface between media and adventitia layers) as the transmural pressure increases from 80 to 160 mmHg. Accordingly, Fig. 7B shows the relative difference of Cauchy stresses as the thickness of IM layer varies in correspondence with Fig. 6. There is a significant increase in $|\frac{{\sigma }_{r}^{3D}-{\sigma }_{r}^{2D}}{{\sigma }_{r}^{3D}}|$, but a relative constant in $|\frac{{\sigma }_{\theta }^{3D}-{\sigma }_{\theta }^{2D}}{{\sigma }_{\theta }^{3D}}|$ with the increase of the transmural pressure and the thickness ratio of the IM layer to the entire vessel wall.

## Discussion

The study demonstrated a 3D analysis of active and passive mechanical properties of coronary arteries in normal directions, based on the experimental measurements^17,18^. A two-layer model was used to compute the transmural distribution of stresses and stretches across the vessel wall, based on 3D active and passive strain energy functions. The major findings are reported as follows: 1) Good agreement is found between 3D theoretical predictions and experimental measurements; 2) There is a gradual decrease of the 1^st^ PK stresses in a sequence of circumferential, axial and radial directions under active and passive states; and 3) The ratio of radial to circumferential Cauchy stresses in the IM layer has the highest value at the intima layer, which is significantly increased by contraction.

### 3D active strain energy function

We previously carried out biaxial mechanical tests in the entire vessel wall and IM layer of porcine RCAs, based on which 2D active and passive models were determined in circumferential and axial directions^17,18^. The present study extends the previous model to 3D active and passive strain energy functions including the radial stress-strain relationship. The GA method is more likely to reach a global minimum than the traditional L-BFGS method (i.e., limited-memory quasi-Newton method for large-scale optimization)^28^. We further enhanced the GA method by estimation of the initial material constants from the M-L method, which significantly reduced the spread of material constants in Tables 1 and 2. The improved GA method has the advantage of accuracy over the L-BFGS method in previous studies^17,18^ albeit it is much more time-consuming. Based on the GA method, the error function for total 1^st^ PK stresses in Eq. [3] was minimized to determine material constants of the 3D active strain energy function (R^2^ > 0.92 in the three normal directions) instead of the error function for active 1^st^ PK stresses in the 2D studies (R^2^ <0.85 in both circumferential and radial directions)^17,18^. Minimizing the error function in Eq. [3] by the proposed GA method significantly improved the accuracy of the optimal fit of experimental measurements to the 3D active strain energy function.

Kassab and his colleagues investigated passive mechanical properties of porcine coronary arteries and reported a larger slope of the circumferential stress-strain relation in the physiological pressure range for the IM layer than the entire vessel wall^26,27,29^, which agrees with Fig. 1A and B. This study further shows larger passive elastic moduli in the physiological pressure range in all three directions of the IM layer than the entire vessel wall, as shown in Figs 1–3. Since SMCs mainly reside in the IM layer, the peak magnitudes of active stress in the IM layer were similar to those in the entire vessel wall (Figs 1A, 2A and 3A vs. Figures 1B, 2B and 3B).

Material constants *b*_1_ to *b*_3_ represent the relative curve width of active stresses as a function of circumferential, axial, and radial stretches, respectively. The sensitivity analysis in Fig. 4 showed that the change of *b*_3_ affects the peak magnitude of the radial active stress and the corresponding stretch while *b*_1_ and *b*_2_ only led to the stretch change in correspondence to the peak value. Similarly, parameters *b*_1_ and *b*_2_ in the 3D active model altered circumferential and axial active stresses, respectively, consistent with the 2D active model despite the absence of radial parameters. Since a previous study only carried out the optimal fit of experimental data in the physiological range^18^, the small values of *b*_1_ and *b*_2_ in the 2D active model referred to the narrower curve width. Furthermore, Tables 1 and 2 showed similar values of *b*_1_ (0.51 ± 0.1 vs. 0.45 ± 0.24) and *b*_2_ (1.06 ± 0.2 vs. 0.83 ± 0.37) in the IM layer and entire vessel wall, but significantly higher values of *b*_3_ (4.29 ± 1.9 vs. 1.89 ± 1.21) in the IM layer than the entire vessel wall, which indicated the radial constraint of the adventitia layer. On the other hand, we found that ${T}_{\theta \theta } > {T}_{zz} > |{T}_{rr}|$ and ${T}_{\theta \theta }^{IM} > {T}_{zz}^{IM} > |{T}_{rr}^{IM}|$ in the active state corresponding to ${b}_{1} < {b}_{2} < {b}_{3}$ and ${b}_{1}^{IM} < {b}_{2}^{IM} < {b}_{3}^{IM}$, respectively. Circumferential 1^st^ PK stresses were approximately 5-fold or more of radial values.

### Transmural distribution of stresses and stretches

A two-layer model was previously developed to compute the transmural distribution of circumferential Cauchy stresses based on the 2D active strain energy function and 3D passive strain energy function^17^. Here, the two-layer model was used to determine the transmural distribution of normal Cauchy stresses in all three directions from 3D active and passive strain energy functions. We selected the axial stretch of 1.3, transmural pressure of 80 mmHg, and wall thickness of 0.22 mm to mimic the physiological state of the RCA, similar to a previous study^17^. Since the opening angle and zero-stress circumferential length of the IM layer were larger than those of the adventitia layer^17^, this led to discontinuous circumferential and radial stretches (despite continuous displacements) at the interface between IM and adventitia layers as well as higher ${\lambda }_{\theta }$ and lower ${\lambda }_{r}$ in the IM layer given that ${\lambda }_{r}=\frac{1}{{\lambda }_{\theta }{\lambda }_{z}}$ at ${\lambda }_{z}$ of 1.3, as shown in Fig. 5C and D. On the other hand, circumferential Cauchy stresses increased significantly from the intima layer to the interface between media and adventitia layers, dropped abruptly at the interface, and increased slightly towards the outer boundary of the adventitia layer. In contrast, absolute values of radial Cauchy stress decreased continuously from the inner to outer boundaries of the entire vessel wall albeit the slope in the IM layer was much higher than that in the adventitia layer. There were the highest radial Cauchy stress and stretch, but the lowest circumferential Cauchy stress and stretch at the inner wall of the IM layer. Hence, it is necessary to consider the mechanical stimuli of both circumferential and radial stresses and stretches to the atherosclerosis-prone intima layer.

K^+^-induced SMC contraction significantly reduced circumferential Cauchy stresses in both IM and adventitia layers, but had relatively negligible effects on radial Cauchy stresses. Microstructure observations have shown that radial tilt angle of SMCs is about 8° and much smaller than tilt angles in other directions^5,30–32^, which supports the present theoretical predictions. The increased ratio of radial to circumferential Cauchy stresses in active state further indicated the significance of radial stresses in the study of vascular remodeling.

### Potential implications for coronary artery mechanics in pressure overload

Wang and Kassab have shown that an increase in opening angle due to acute pressure increase shifts excessive circumferential Cauchy stresses from the IM layer to the adventitia layer^33,34^. Here, we used the two-layer model with 3D active and passive strain energy functions to compute the transmural distribution of circumferential and radial active stresses. Given the uniform circumferential stress hypothesis^35,36^, the transmural pressure and wall thickness in pressure overload were set to 160 mmHg and 0.44 mm (twofold increase from those at the normal state), respectively. This significantly increased the ratio of radial to circumferential stresses (an increase from $\frac{1}{7}$ to $\frac{1}{2}$ at the intima layer) owing to the constant circumferential stress and the increased radial Cauchy stress that is proportional to the increase in blood pressure, as shown in Fig. 6. Moreover, we simulated the transmural distribution of active stresses with the increased ratio of IM to Adventitia layer thicknesses, which further increased the ratio of radial to circumferential stresses. This illustrates the importance of radial stresses to coronary artery mechanics in pressure overload.

### A comparison with the 2D active strain energy function

Wang *et al*. showed the 3D passive mechanical properties in the IM layer and entire vessel wall of porcine coronary arteries and indicated that the 3D passive model served as a foundation for formulation of layer-specific boundary value problems^26^. Here, we showed the 3D active mechanical properties in the IM layer. In comparison with the 2D active model^17,18^, the 3D active model revealed the following features: 1) The fit to experimental measurements was improved; 2) The radial term significantly affected the peak magnitude of the radial active stress and the corresponding stretch, but only led to a slight stretch change in correspondence to the peak circumferential active stress (Fig. 4; 3) ${\sigma }_{\theta }^{3D} < {\sigma }_{\theta }^{2D}$ and $|{\sigma }_{r}^{3D}| > |{\sigma }_{r}^{2D}|$ for the transmural distribution of Cauchy stresses determined by the two-layer model with the 2D or 3D active strain energy function in the IM layer as well as the same 3D passive strain energy function in the entire vessel wall; and 4) $|\frac{{\sigma }_{r}^{3D}-{\sigma }_{r}^{2D}}{{\sigma }_{r}^{3D}}|$ at the interface between media and adventitia layers increased significantly when the transmural pressure and the thickness ratio of the IM layer to the entire vessel wall increased from normal to pressure-overload states (<1% at the normal state and >10% at the pressure-overload state, as shown in Fig. 7) despite negligible changes of $|\frac{{\sigma }_{\theta }^{3D}-{\sigma }_{\theta }^{2D}}{{\sigma }_{\theta }^{3D}}|$ (~3% in both normal and pressure-overload states). This shows the advantage of the 3D active strain energy function over the 2D active model in a previous study^17^ in the study of vascular mechanics under pressure overload (i.e., mimic hypertension).

### Critique of the study

Although this study investigated 3D active and passive mechanical properties in normal directions, the stresses in shear directions were neglected. Multiple CADs (e.g., atherosclerosis, aneurysm, and vessel dissection) change both morphometry and components of vessel wall and affect the distribution of stresses in all three directions. The altered ratio of radial to circumferential stresses may result in the relief or deterioration of these CADs. Hence, the following studies should include stresses in all normal and shear directions of healthy and diseased vessels.

## Conclusions

Material constants of 3D active and passive strain energy functions were determined in the entire vessel wall and IM layers of porcine RCAs. A 3D mechanical analysis demonstrated a gradual decrease of active and passive 1^st^ PK stresses in the order of circumferential, axial and radial directions. Moreover, a two-layer model showed that Cauchy stresses were discontinuous in the circumferential direction, but continuous in the radial direction. The ratio of radial to circumferential Cauchy stress was the highest at the inner boundary of the IM layer, which significantly increased in contraction. This study enhances our understanding the distribution of intramural stresses in vessel wall.

## Electronic supplementary material

Supplementary Information 

## References

[1] Piccolo R, Giustino G, Mehran R, Windecker S (2015). Stable coronary artery disease: revascularisation and invasive strategies. Lancet.

[2] Pfisterer ME, Zellweger MJ, Gersh BJ (2010). Management of stable coronary artery disease. Lancet.

[3] Chiu JJ, Chien S (2011). Effects of disturbed flow on vascular endothelium: pathophysiological basis and clinical perspectives. Physiol Rev.

[4] Green DJ, Hopman MT, Padilla J, Laughlin MH, Thijssen DH (2017). Vascular Adaptation to Exercise in Humans: Role of Hemodynamic Stimuli. Physiol Rev.

[5] Chen H, Kassab GS (2016). Microstructure-based biomechanics of coronary arteries in health and disease. J Biomech.

[6] Waffenschmidt T (2016). Towards the modelling of ageing and atherosclerosis effects in ApoE(-/-) mice aortic tissue. J Biomech.

[7] Sommer G (2016). Mechanical strength of aneurysmatic and dissected human thoracic aortas at different shear loading modes. J Biomech.

[8] Wagenseil JE, Mecham RP (2009). Vascular extracellular matrix and arterial mechanics. Physiol Rev.

[9] Holzapfel GA, Ogden RW (2010). Modelling the layer-specific three-dimensional residual stresses in arteries, with an application to the human aorta. Journal of the Royal Society Interface.

[10] Rachev, A. & Hayashi, K. *Theoretical study of the effects of vascular smooth muscle contraction on strain and stress distributions in arteries*. (Eyre & Spottiswoode, 1999). 10.1114/1.191 10468230

[11] Greenwald SE, Moore JE, Rachev A, Kane TP, Meister JJ (1997). Experimental investigation of the distribution of residual strains in the artery wall. J Biomech Eng.

[12] Rachev A (1997). Theoretical study of the effect of stress-dependent remodeling on arterial geometry under hypertensive conditions. J Biomech.

[13] Cornelissen AJ, Dankelman J, VanBavel E, Spaan JA (2002). Balance between myogenic, flow-dependent, and metabolic flow control in coronary arterial tree: a model study. Am J Physiol Heart Circ Physiol.

[14] Carlson BE, Secomb TW (2005). A theoretical model for the myogenic response based on the length-tension characteristics of vascular smooth muscle. Microcirculation.

[15] Humphrey JD (2008). Mechanisms of arterial remodeling in hypertension: coupled roles of wall shear and intramural stress. Hypertension.

[16] Humphrey, J. D. *Cardiovascular Solid Mechanics*. (Springer-Verlag, 2002).

[17] Huo Y, Zhao X, Cheng Y, Lu X, Kassab GS (2013). Two-layer model of coronary artery vasoactivity. J Appl Physiol (1985).

[18] Huo Y, Cheng Y, Zhao X, Lu X, Kassab GS (2012). Biaxial vasoactivity of porcine coronary artery. Am J Physiol Heart Circ Physiol.

[19] Wagner HP, Humphrey JD (2011). Differential passive and active biaxial mechanical behaviors of muscular and elastic arteries: basilar versus common carotid. J Biomech Eng.

[20] Fung YC, Drucker DC (1966). Foundation of Solid Mechanics. Journal of Applied Mechanics.

[21] Fung YC, Fronek K, Patitucci P (1979). Pseudoelasticity of arteries and the choice of its mathematical expression. American Journal of Physiology.

[22] Debes JC, Fung YC (1995). Biaxial mechanics of excised canine pulmonary arteries. American Journal of Physiology - Heart and Circulatory Physiology.

[23] Siepmann P, Martin CP, Vancea I, Moriarty PJ, Krasnogor N (2007). A genetic algorithm approach to probing the evolution of self-organized nanostructured systems. Nano letters.

[24] Ogden RW (1984). Non-linear elastic deformations. Journal of Applied Mechanics.

[25] Holzapfel GA, Ogden RW (2010). Constitutive modelling of arteries. Proceedings of the Royal Society A: Mathematical, Physical and Engineering Science.

[26] Wang C, Garcia M, Lu X, Lanir Y, Kassab GS (2006). Three-dimensional mechanical properties of porcine coronary arteries: a validated two-layer model. Am J Physiol Heart Circ Physiol.

[27] Pandit A, Lu X, Wang C, Kassab GS (2005). Biaxial elastic material properties of porcine coronary media and adventitia. Am J Physiol Heart Circ Physiol.

[28] Liu DC, Nocedal J (1989). On the Limited Memory Bfgs Method for Large-Scale Optimization. Math Program.

[29] Lu X, Pandit A, Kassab GS (2004). Biaxial incremental homeostatic elastic moduli of coronary artery: two-layer model. Am J Physiol Heart Circ Physiol.

[30] Chen H (2013). Microstructural constitutive model of active coronary media. Biomaterials.

[31] Chen H, Guo X, Luo T, Kassab GS (2016). A validated 3D microstructure-based constitutive model of coronary artery adventitia. J Appl Physiol (1985).

[32] Luo T, Chen H, Kassab GS (2016). 3D Reconstruction of Coronary Artery Vascular Smooth Muscle Cells. PLoS One.

[33] Wang C, Guo X, Kassab GS (2009). A new observation on the stress distribution in the coronary artery wall. J Biomech Eng.

[34] Wang C, Kassab GS (2009). Increase in opening angle in hypertension off-loads the intimal stress: a simulation study. J Biomech Eng.

[35] Kassab GS (2002). Remodelling of the left anterior descending artery in a porcine model of supravalvular aortic stenosis. J Hypertens.

[36] Huo Y, Kassab GS (2012). Compensatory remodeling of coronary microvasculature maintains shear stress in porcine left-ventricular hypertrophy. J Hypertens.

