# Supplementary material for: Passive and Active Triaxial Wall Mechanics in a Two-Layer Model of Porcine Coronary Artery
Source: Sci Rep. 2017 Oct 24;7:13911. doi: 10.1038/s41598-017-14276-1 (PMC5655692; doi:10.1038/s41598-017-14276-1)
Supplement: Supplementary file 1 — Supplementary Information [file 41598_2017_14276_MOESM1_ESM.pdf]

Supplementary Materials

Passive and Active Triaxial Wall Mechanics in a Two-Layer Model of Porcine Coronary Artery

Yuan Lu[1], Hao Wu[2], Jiahang Li[2], Yanjun Gong[1], Jiahui Ma[4], Ghassan S. Kassab[3], Yong Huo[1], Wenchang Tan[2,5,6], and Yunlong Huo[2,6]

[1]Department of Cardiology, Peking University First Hospital, Beijing, China
[2]Department of Mechanics and Engineering Science, College of Engineering, Peking University, Beijing, China
[3]California Medical Innovations Institute, San Diego, USA
[4]School of Public Health, Peking University, Beijing, China
[5]Shenzhen Graduate School, Peking University, Shenzhen, China
[6]PKU-HKUST Shenzhen-Hongkong Institution, Shenzhen, China

Running Title: Active and passive mechanical properties of coronary artery

APPENDIX A

Biomechanical principles: In cylindrical coordinate with R, Z, Θ for stress-free state and r, z, θ for no-load and loaded states, we obtain the following expression:

$$r = r(R), \qquad \theta = \frac{\pi}{\pi - \varphi}\Theta = \chi\Theta, \qquad Z = \lambda_z z \qquad \textbf{(A1)}$$

where $\chi = \frac{\pi}{\pi-\varphi}$ is a parameter that characterizes the deformation from stress-free to loaded states depending on the opening angle φ. As the shear deformation is neglected, deformation gradient and green strain tensor are written as:

$$F = \begin{bmatrix} \lambda_r & 0 & 0 \\ 0 & \lambda_\theta & 0 \\ 0 & 0 & \lambda_z \end{bmatrix} = \begin{bmatrix} \frac{dr}{dR} & 0 & 0 \\ 0 & \frac{\chi r}{R} & 0 \\ 0 & 0 & \lambda_z \end{bmatrix} \qquad \text{(A2)}$$

$$E = \frac{1}{2}[F^T F - 1] = \begin{bmatrix} E_{rr} & 0 & 0 \\ 0 & E_{\theta\theta} & 0 \\ 0 & 0 & E_{zz} \end{bmatrix} =$$

$$\begin{bmatrix} \frac{1}{2}(\lambda_r^2 - 1) & 0 & 0 \\ 0 & \frac{1}{2}(\lambda_\theta^2 - 1) & 0 \\ 0 & 0 & \frac{1}{2}(\lambda_z^2 - 1) \end{bmatrix} \qquad \text{(A3)}$$

where $E_{\theta\theta}$, E_{zz} and E_{rr} are circumferential, axial and radial Green strains, respectively; $\lambda_\theta = \frac{l}{l_0} = \sqrt{2E_{\theta\theta} + 1}$, $\lambda_z = \frac{L}{L_0} = \sqrt{2E_{zz} + 1}$, and $\lambda_r = \frac{1}{\lambda_\theta \lambda_z}$ (given the material incompressibility, i.e., $\lambda_r \lambda_\theta \lambda_z = 1$) represent the corresponding stretch ratios (l and l_0 are the circumferential lengths in loaded and zero-stress states; and L and L_0 are the axial lengths in loaded and no-load states). The material incompressibility results in the following equation:

$$R = \sqrt{(r^2 - r_i^2)\chi\lambda_z + R_i^2} \qquad \textbf{(A4)}$$

Strain energy function: The 3D passive and active strain energy functions ($W_{passive}$ and W_{active}, respectively) are written as:

$$W_{passive} = \frac{1}{2} C_1 \left[e^Q - 1 \right]$$
$$W_{active} = C_2 \left[Erf\left(\frac{\lambda_\theta}{b_1} + \frac{\lambda_z}{b_2} + \frac{\lambda_r}{b_3} - b^{'} \right) - 1 \right] \qquad \textbf{(A5)}$$

where $Q = a_1 E_{\theta\theta}^2 + a_2 E_{zz}^2 + a_3 E_{rr}^2 + 2a_4 E_{\theta\theta} E_{zz} + 2a_5 E_{zz} E_{rr} + 2a_6 E_{rr} E_{\theta\theta}$ and $b^{'} = \frac{b_4}{b_1} + \frac{b_5}{b_2} + \frac{b_6}{b_3}$; C_1, C_2, $a_1 - a_6$ and $b_1 - b_6$ are material constants; and $Erf(X)$ is the Gauss error function. $W_{passive}$ refers to $W_{passive}^{IM}$ or $W_{passive}^{A}$ while W_{active} represents W_{active}^{IM} because K[+]-induced active strain energy function is only applied to the intima-media layer with SMCs.

The 3D passive and active 2nd PK stresses are written as:

$$S_{\theta\theta_{passive}} = \frac{\partial W_{passive}}{\partial E_{\theta\theta}} = C_1 \left(a_1 E_{\theta\theta} + a_4 E_{zz} + a_6 E_{rr} \right) \left(e^Q \right)$$
$$S_{zz_{passive}} = \frac{\partial W_{passive}}{\partial E_{zz}} = C_1 \left(a_2 E_{zz} + a_4 E_{\theta\theta} + a_5 E_{rr} \right) \left(e^Q \right)$$
$$S_{rr_{passive}} = \frac{\partial W_{passive}}{\partial E_{rr}} = C_1 \left(a_3 E_{rr} + a_5 E_{zz} + a_6 E_{\theta\theta} \right) \left(e^Q \right)$$
$$S_{\theta\theta_{active}} = \frac{\partial W_{active}}{\partial E_{\theta\theta}} = \frac{2C_2}{b_1 \sqrt{\pi} \sqrt{2E_{\theta\theta} + 1}} \exp\left[-\left(\frac{\sqrt{2E_{\theta\theta}+1}}{b_1} + \frac{\sqrt{2E_{zz}+1}}{b_2} + \frac{\sqrt{2E_{rr}+1}}{b_3} - b^{'} \right)^2 \right] \qquad \textbf{(A6)}$$
$$S_{zz_{active}} = \frac{\partial W_{active}}{\partial E_{zz}} = \frac{2C_2}{b_2 \sqrt{\pi} \sqrt{2E_{zz} + 1}} \exp\left[-\left(\frac{\sqrt{2E_{\theta\theta}+1}}{b_1} + \frac{\sqrt{2E_{zz}+1}}{b_2} + \frac{\sqrt{2E_{rr}+1}}{b_3} - b^{'} \right)^2 \right]$$
$$S_{rr_{active}} = \frac{\partial W_{active}}{\partial E_{rr}} = \frac{2C_2}{b_3 \sqrt{\pi} \sqrt{2E_{rr} + 1}} \exp\left[-\left(\frac{\sqrt{2E_{\theta\theta}+1}}{b_1} + \frac{\sqrt{2E_{zz}+1}}{b_2} + \frac{\sqrt{2E_{rr}+1}}{b_3} - b^{'} \right)^2 \right]$$

The 3D 1st PK stresses at passive and active states are written as:

$$
\begin{aligned}
T_{\theta\theta_{passive}} &= \lambda_\theta S_{\theta\theta_{passive}} = C_1\lambda_\theta\left(a_1E_{\theta\theta}+a_4E_{zz}+a_6E_{rr}\right)\left(e^Q\right)\\
T_{zz_{passive}} &= \lambda_z S_{zz_{passive}} = C_1\lambda_z\left(a_2E_{zz}+a_4E_{\theta\theta}+a_5E_{rr}\right)\left(e^Q\right)\\
T_{rr_{passive}} &= \lambda_r S_{rr_{passive}} = C_1\lambda_r\left(a_3E_{rr}+a_5E_{zz}+a_6E_{\theta\theta}\right)\left(e^Q\right)\\
T_{\theta\theta_{active}} &= \lambda_\theta S_{\theta\theta_{active}} = \frac{2C_2}{b_1\sqrt{\pi}}\exp\left[-\left(\frac{\sqrt{2E_{\theta\theta}+1}}{b_1}+\frac{\sqrt{2E_{zz}+1}}{b_2}+\frac{\sqrt{2E_{rr}+1}}{b_3}-b'\right)^2\right]\\
T_{zz_{active}} &= \lambda_z S_{zz_{active}} = \frac{2C_2}{b_2\sqrt{\pi}}\exp\left[-\left(\frac{\sqrt{2E_{\theta\theta}+1}}{b_1}+\frac{\sqrt{2E_{zz}+1}}{b_2}+\frac{\sqrt{2E_{rr}+1}}{b_3}-b'\right)^2\right]\\
T_{rr_{active}} &= \lambda_r S_{rr_{active}} = \frac{2C_2}{b_3\sqrt{\pi}}\exp\left[-\left(\frac{\sqrt{2E_{\theta\theta}+1}}{b_1}+\frac{\sqrt{2E_{zz}+1}}{b_2}+\frac{\sqrt{2E_{rr}+1}}{b_3}-b'\right)^2\right]
\end{aligned}
\qquad \textbf{(A7)}
$$

Equations (A7) and (A8) were used to determine Piola-Kirchhoff stresses.

APPENDIX B

The equilibrium equation in cylindrical coordinate is given as:

$$\frac{\partial \sigma_r}{\partial r} + \frac{\sigma_r - \sigma_\theta}{r} = 0 \quad \textbf{(B1)}$$

where σ_r and σ_θ are radial and circumferential Cauchy stresses, respectively. The passive Cauchy stress tensor is written as:

$$\sigma_{passive} = F \cdot \frac{\partial W_{passive}}{\partial E} \cdot F^T - PI \quad \textbf{(B2)}$$

where P is the unknown Lagrange multiplier which needs to be determined form boundary conditions. The K^+-induced 3D active strain energy function was used to model the fully contraction of the intima-media layer, i.e., $W_{total}^{IM} = W_{passive}^{IM} + W_{active}^{IM}$. The total Cauchy stress in the intima-media layer is given by

$$\sigma_{total} = F \cdot \frac{\partial \left(W_{passive}^{IM} + W_{active}^{IM} \right)}{\partial E} \cdot F^T - PI \quad \textbf{(B3)}$$

The adventitia layer does not contribute to the active contraction of the artery and thus only the passive strain energy is considered in the adventitia layer, i.e., $W_{total}^{A} = W_{passive}^{A}$.

The boundary conditions at the inner and outer surfaces of vessel wall are:

$$\sigma_r|_{r=r_i^{IM}} = -p \quad \text{and} \quad \sigma_r|_{r=r_o^{A}} = 0 \quad \textbf{(B4)}$$

where p equals to 80 mmHg. Subscripts 'i' and 'o' refer to inner and outer boundaries. An additional boundary condition is the stress balance at the interface between intima-media and adventitia layers:

$$\sigma_r|_{r=r_o^{IM}} = \sigma_r|_{r=r_i^{A}} \quad \textbf{(B5)}$$

Solving Eq. (B1) with boundary conditions, radial, circumferential, and axial stress components in the intima-media layer are given by:

$$\begin{cases} \sigma_r^{IM} = \int_{r_i^{IM}}^{r} \left(\lambda_\theta^2 \frac{\partial W^{IM}}{\partial E_{\theta\theta}} - \lambda_r^2 \frac{\partial W^{IM}}{\partial E_{rr}} \right) \frac{1}{r} dr - p \\ \sigma_\theta^{IM} = \sigma_r^{IM} + \lambda_\theta^2 \frac{\partial W^{IM}}{\partial E_{\theta\theta}} - \lambda_r^2 \frac{\partial W^{IM}}{\partial E_{rr}} \\ \sigma_z^{IM} = \sigma_r^{IM} + \lambda_z^2 \frac{\partial W^{IM}}{\partial E_{zz}} - \lambda_r^2 \frac{\partial W^{IM}}{\partial E_{rr}} \end{cases} \quad \textbf{(B6)}$$

From Eq. (B5), we can obtain:

$$\sigma_r|_{r=r_i^A} = \sigma_r|_{r=r_o^{IM}} = \int_{r_i^{IM}}^{r_o^{IM}} \left(\lambda_\theta^2 \frac{\partial W^{IM}}{\partial E_{\theta\theta}} - \lambda_r^2 \frac{\partial W^{IM}}{\partial E_{rr}} \right) \frac{1}{r} dr - p \quad \textbf{(B7)}$$

Solving Eq. (B1) with boundary conditions, radial, circumferential, and axial stress components in the adventitia layer are given by:

$$\begin{cases} \sigma_r^{A} = \int_{r_i^{A}}^{r} \left(\lambda_\theta^2 \frac{\partial W^{A}}{\partial E_{\theta\theta}} - \lambda_r^2 \frac{\partial W^{A}}{\partial E_{rr}} \right) \frac{1}{r} dr + \int_{r_i^{IM}}^{r_o^{IM}} \left(\lambda_\theta^2 \frac{\partial W^{IM}}{\partial E_{\theta\theta}} - \lambda_r^2 \frac{\partial W^{IM}}{\partial E_{rr}} \right) \frac{1}{r} dr - p \\ \sigma_\theta^{A} = \sigma_r^{A} + \lambda_\theta^2 \frac{\partial W^{A}}{\partial E_{\theta\theta}} - \lambda_r^2 \frac{\partial W^{A}}{\partial E_{rr}} \\ \sigma_z^{A} = \sigma_r^{A} + \lambda_z^2 \frac{\partial W^{A}}{\partial E_{zz}} - \lambda_r^2 \frac{\partial W^{A}}{\partial E_{rr}} \end{cases} \quad \textbf{(B8)}$$

Given the appropriate strain energy functions, Equations (B1-B8) were used to solve the transmural stress-strain relationship from stress-free to loaded vessel wall at both passive and active states.
